# A Day/Night Leader-Following Method Based on Adaptive Federated Filter for Quadruped Robots

**DOI:** 10.3390/biomimetics8010020

**Published:** 2023-01-04

**Authors:** Jialin Zhang, Jiamin Guo, Hui Chai, Qin Zhang, Yibin Li, Zhiying Wang, Qifan Zhang

**Affiliations:** 1 School of Control Science and Engineering, Shandong University, Jinan 250100, China; 2 Robotics Research Center, Shandong University, Jinan 250100, China; 3 School of Electrical Engineering, University of Jinan, Jinan 250024, China

**Keywords:** sensor fusion, vision navigation, quadruped robot

## Abstract

The quadruped robots have superior adaptability to complex terrains, compared with tracked and wheeled robots. Therefore, leader-following can help quadruped robots accomplish long-distance transportation tasks. However, long-term following has to face the change of day and night as well as the presence of interference. To solve this problem, we present a day/night leader-following method for quadruped robots toward robustness and fault-tolerant person following in complex environments. In this approach, we construct an Adaptive Federated Filter algorithm framework, which fuses the visual leader-following method and the LiDAR detection algorithm based on reflective intensity. Moreover, the framework uses the Kalman filter and adaptively adjusts the information sharing factor according to the light condition. In particular, the framework uses fault detection and multisensors information to stably achieve day/night leader-following. The approach is experimentally verified on the quadruped robot SDU-150 (Shandong University, Shandong, China). Extensive experiments reveal that robots can identify leaders stably and effectively indoors and outdoors with illumination variations and unknown interference day and night.

## 1. Introduction

Quadruped robots are often used to perform tasks in the field [1] because of their demonstrated adaptability to rugged terrain compared with the wheeled and tracked [2,3]. However, leader-following in the field environments is a problem that quadruped robots must solve to complete tasks like long-distance field transportation [4]. First, the leader must be consistently and accurately identified in real-time using various sensors to achieve autonomous following [5,6]. The quadruped robot is then controlled to follow the leader.

Leader-following of quadruped robots is a current research hotspot. With the development of computer vision, the accuracy of visual person detection has been significantly improved. LiDAR has also been applied to pedestrian detection and achieves a good effect. At present, researchers use LED lighting and visual detection to achieve nighttime leader-following [7,8]. Meanwhile, the motion control capability of quadruped robots has been significantly enhanced, which makes long-distance field leader-following possible [1,9].

The development, as mentioned above, effectively solves the accuracy of leader-following. Still, there is not enough attention to leader-following’s stability and fault tolerance for quadruped robots in the field, especially at night. As a result, the leader-following for quadruped robots still has several problems: (1) Stable detection is still a challenge due to the high-frequency vibration of quadruped robots, and the influence of rough terrain. (2) It is tough for a single sensor to deal with complex and various environments. (3) The long-distance leader-following at night cannot totally be solved due to the limited lighting distance of LED light, especially when the energy of quadruped robots is constrained.

To solve the above questions, a day/night leader-following approach for quadruped robots is proposed. We build a visual leader-following framework [10], using the Single Shot MultiBox Detector (SSD), Kernelized Correlation Filters (KCF) and Person Reidentification (Re-ID), which can stably detect person. Concurrently, a LiDAR-based leader-following framework is built, which can effectively detect the leader [11]. The algorithm set out in this study overcomes the problem of false recognition caused by light interference and some objects with high reflectivities, such as a license plate, especially at night. Our main contributions to this study can be summarized as follows:

- We build a leader-following system including person detection, communication module, and motion control module. This system enables the quadruped robot to follow a leader in real time.
- We propose an Adaptive Federated Filter algorithm framework, which can adaptively adjust the information sharing factors according to light conditions. The algorithm combines visual and LiDAR-based detection frameworks, which helps quadruped robots achieve day/night leader-following.
- We establish a fault detection and isolation algorithm that dramatically improves the stability and robustness of day/night leader-following. In this algorithm, we fully use multisensors information from sensors and detection algorithms, which can adapt to high-frequency vibrations, illumination variations and interference from reflective materials.

The proposed approach achieves a long-term and stable day/night leader-following for quadruped robots with superiority in handling light variations and interference.

The remainder of this study is organized as follows. Relevant research works are discussed in Section 2. In Section 3, our algorithm is introduced in detail. Experiments are presented in Section 4, and Section 5 concludes this study.

## 2. Related Works

Innovative studies have been conducted using different sensors to locate the leader in real time. Depending on the sensor, the solutions can be divided into three categories: visual leader-following, LiDAR-based leader-following, and multisensor combination.

A popular person identification method uses visual detection to capture RGB and depth information and then identify the leader by detecting features like human body color and shape. Visual trackers [12] have been applied to leader-following schemes because of their high accuracy. K. Zhang et al. [13], and J. Guo et al. [14] applied a real-time 3D perception system to predict the locomotion intent of humans based on RGB-D. M. Gupta et al. [6] constructed a novel visual method for a human-following mobile robot, which enables robots to be around humans all the time at home. However, current visual approaches applied to quadruped robots are easily limited by light in the field environment. The effect is poor in low light, strong light, light transformation, and darkness. To solve the above problem, pioneering research has been conducted to adapt to the drastic light changes in the field environment, especially at night. For instance, M. Bajracharya et al. [7] used LED light for vegetation and negative obstacle detection at night in the Legged Squad Support System (LS3). In the DARPA Subterranean Challenge Competition, most quadruped robots, including champion ANYmal, use LED illumination and camera perception to effectively detect persons and objects in the darkness [8].

LiDAR is commonly used to follow a person by detecting the human body. A. Leigh et al. [15], and E. Jung et al. [16] built a person tracking system using 2D LiDAR to achieve the human-following and obstacle avoidance. J. Yuan et al. [17] presented a human-following method based on 3D LiDAR with a mobile robot in indoor environments. Currently, the LiDAR-based leader-following approach based on reflection intensity is more adaptable to complex scenes than the algorithm based on personal characteristics, and this approach is less affected by vibration [18]. However, this method also has the disadvantage of being easily disturbed by reflective metal materials [11].

Multisensors are deployed for person detection to overcome the limitation of obtaining environmental information from a single sensor. X. Meng et al. [19] fused data from LiDAR and a camera for detection and tracking. C. Wang et al. [20], and H. Zhao et al. [21] combined an RGB-D camera with a laser to construct maps for efficient object search. In mobile robot localization, R. Voges et al. [22] assigned the distance measured by LiDAR to the detected visual features to improve recognition stability. Wang et al. [23] present a real-time robotic 3-D human tracking system that combines a monocular camera with an ultrasonic sensor. M. Perdoch et al. [24] constructed a marker tracking system that uses near-infrared cameras, retroreflective markers, and LiDAR to allow a particular user to designate himself as the robot’s leader and guide the robot along the desired path. K. Eckenhoff et al. [25] designed a visual–inertial navigation system for mobile robots to achieve efficient 3D motion tracking.

Quadruped robots can use sensors are various. Reducing the fusion error rate and uncertainty is the main problem of sensor fusion. Kalman Filters, Markov chain, and Monte Carlo method are common techniques used nowadays, which cannot fully solve the above problem [18]. N. Dwek et al. [26] designed sensor fusion and outlier detection to improve robust Ultra Wide Band positioning performance. T. Ji et al. [27] used fault detection for navigation failure in agricultural environments to combat sensor occlusion in cluttered environments.

Our method uses the multisensors fusion approach. We fuse the LiDAR-based leader-following based on reflection intensity and the visual detection algorithm based on the tracker. Our fusion framework improves the fault tolerance of pedestrian detection and helps quadruped robots detect leaders at night. Additionally, the leader misidentification rate is effectively reduced by using multisensors information. We also design a fault detection algorithm to achieve a more stable leader-following at night.

## 3. Methods

Federal filtering is a decentralized filtering algorithm. Compared with centralized filtering, it has the advantage of simple implementation and flexible information sharing. Therefore, we adopt the federated filtering algorithm to overcome the problems of large calculations and poor fault tolerance in multisensor fusion.

**Figure 1:**
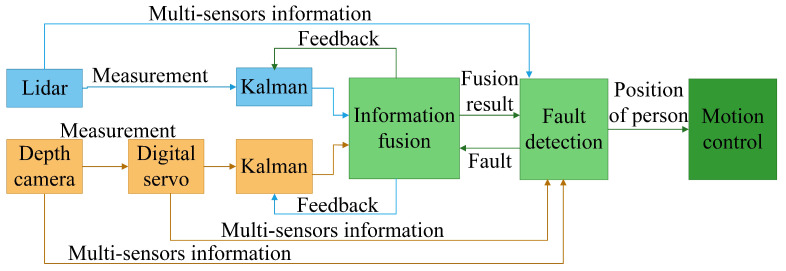
Adaptive federated filter framework, including information update, information fusion, information sharing factors, feedback resetting, fault detection and isolation.

The proposed federated filtering framework is designed to fuse the location information of the leader. This framework adopts an adaptive information factor and uses multisensors information to check the fault. The whole framework adopts a two-stage design. The first stage is a subfilter using Kalman filtering, which is used for local prediction and estimation to predict the person’s position by a single sensor. The second stage is the main filter, which is used for optimal global estimation to fuse the information from the subfilter. Figure 1 shows the proposed federated filtering framework. The framework includes the following parts:

### 3.1. Information Update

The subfilter applies Kalman filtering on the leader position information collected by the LiDAR and visual algorithms, respectively.

The subfilter updates the prediction of the leader position X(k/k−1) in the current frame:

(1) X(k/k−1)=AX(k−1/k−1)+BU(k)

(2) $$P\left(k/k-1\right)=AP\left(k-1/k-1\right)A^{\prime }+Q$$

where (1) calculates the prediction of the person position X(k/k−1) in the current frame according to the estimation of the leader position X(k−1/k−1) in the previous frame. (2) gets the covariance matrix P(k/k−1) corresponding to the prediction of leader position X(k/k−1). In (1) and (2), *A* is the state transfer matrix, *B* is the observation matrix and $A^{\prime }$ is the transpose matrix of *A*.

The subfilter updates the estimation of the leader position X(k/k−1) in the current frame:

(3) X(k/k)=X(k/k−1)+Kg(k)[z(k)−Hx(k/k−1)]

(4) P(k/k)=(1−Kg(k)H)P(k/k−1)

(5) $$Kg\left(k\right)=P\left(k/k-1\right)H^{\prime }/\left(HP\left(k/k-1\right)H^{\prime }+R\right)$$

where (3) calculates the optimal estimation of the leader position X(k/k) in the current frame according to the prediction X(k/k−1) and measurement z(k) of the leader position in the current frame. (4) obtains the covariance matrix P(k/k) corresponding to the optimal estimation of person position, and (5) figures out the Kalman gain Kg(k).

### 3.2. Information Fusion

Each subfilter estimates the leader position from a single sensor. These estimations $X_{i}$ and the corresponding covariance matrices $P_{ii}$ are fused in the main filter.

(6) $$P_{g}^{-1}=\sum_{i=1}^{N}P_{ii}^{-1}$$

(7) $$X_{g}=P_{g}^{-1}\left(\sum_{i=1}^{N}P_{ii}^{-1}X_{i}\right)$$

$P_{g}$ is the covariance matrix corresponding to $X_{g}$ in (6). $X_{g}$ is the optimal estimation of the leader position under multisensors in (7). $X_{i}$ is the optimal estimation of the leader position under a single sensor. $P_{ii}$ is the covariance matrix corresponding to $X_{i}$. $X_{i}$ and $P_{ii}$ are computed in the information update step.

### 3.3. Information Sharing Factor

The difference in the information sharing factors determines the structure and performance of the federated filtering, and the setting of the factor must meet the principle of conservation of information distribution:

(8) $$\beta_{m}+\sum_{i=1}^{n}\beta_{i}=1$$

where $\beta_{i}$ is the information sharing factor of the main filter, $\beta_{i}$ is the information sharing factor of the subfilter. The information sharing factor is used to adjust the feedback of the main filter for the subfilters. Therefore, smaller $\beta_{i}$ increases the use of the measurement and reduces the effect of the faulty subfilter.

In this study, we judge the lighting conditions based on the HSV (Hue, Saturation, Value) color space to achieve an adaptive information sharing factor. The HSV color space can effectively reflect the lighting conditions. When the illumination is limited, the reliability of the visual approach decreases. Therefore, our method adaptively adjusts the information sharing factor according to the HSV color space to change the weight of visual information, improving leader detection’s robustness. (9) constructs the relationship between value and information sharing factor.

(9) $$\begin{array}{c} \beta_{m}=\left\{\begin{array}{cc} y_{1}, & \text{if}\;V\in [0,\alpha_{1}]\\ y_{2}, & \text{if}\;V\in [\alpha_{1},\alpha_{2}]\\ y_{3}, & \text{if}\;V\in [\alpha_{2},\alpha_{3}] \end{array}\right. \end{array}$$

$y_{1}$, $y_{2}$, $y_{3}$, $\alpha_{1}$, $\alpha_{2}$ and $\alpha_{3}$ are the empirical values obtained in the experiments, which are related to the performance of the detection algorithm. The specific value is obtained in the experiments in Section 3.

### 3.4. Feedback Resetting

The main filter resets the information of the subfilter. The subfilter obtains the estimation of the person position $P_{i}^{\wedge }$ in the previous frame, and the corresponding covariance matrix $P_{i}^{\wedge }$ and noise matrix $Q_{i}$. The equation is as follows:

(10) $$P_{i}^{\wedge }=\beta_{i}^{-1}P_{g}$$

(11) $$Q_{i}=\beta_{i}^{-1}Q_{g}$$

(12) $$X_{i}^{\wedge }=X_{i}$$

$Q_{g}$ is the noise matrix of the main filter in (11).

### 3.5. Fault Detection and Isolation

We use fault detection method to check whether the subfilter is faulty. After the fault is discovered, we isolate the faulty subfilter to avoid affecting the entire system. Sometimes traditional method like residual method cannot effectively detect sensor faults, so we combine multisensors information to build robust fault detection and isolation system. The visual algorithm can calculate the number of persons in the image, and the digital servo can calculate the rotation angle of the camera. We also recorded the result of visual and LiDAR-based detection in the past. The proposed approach uses this multisensors information for fault detection to achieve more reliable and stable leader-following day and night. The fault detection and isolation systerm can handle tracking failures caused by vibration, illumination variations and interference from strong reflectors. The algorithm is presented in Algorithm 1.
**Algorithm 1: Fault detection and isolation****Input:** measurement of motor, the person position detected by LiDAR algorithm, the number of persons detected by the visual algorithm**Output:** Robot motion control parameters1:  **for** t = 2, 3, …do;2:  **save** past motor angle, LiDAR angle;3:  **if** ((measure - estimate >x) ||((LiDAR angle == 0) && (past LiDAR angle == 0)))4:   {LiDAR fail;5:   LiDAR angle = motor angle;}6:  **if** ((camera people num == 0) && (past motor == const))7:   {camera fail;8:   motor angle = LiDAR angle;}9:  **target angle** = Adaptive Federated Filter(LiDAR angle, motor angle);10:      **motion control parameters** = target angle;11:      **end**

## 4. Experiments

### 4.1. Experimental Setup

This study realizes reliably leader-following day and night. We conducted experiments on the quadruped robot SDU-150 to testify to the proposed approach.

**Figure 2:**
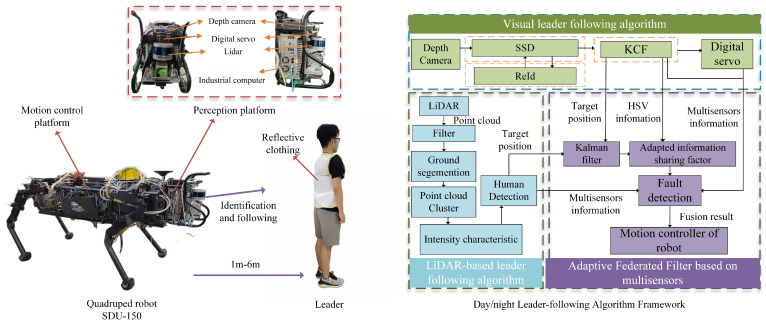
Quadruped robot SDU-150 (Shandong University, Jinan, China) is composed of perception and motion control platforms. The quadruped robot perception platform reveals the positional relationship between the depth camera and the LiDAR. The leader wears reflective clothing and maintains a distance of 1m to 6m from robot. The day/night leader-following algorithm framework includes visual leader-following, LiDAR-based leader-following, and Adaptive Federated Filter.

Figure 2 shows the positional relationship between the depth camera and the LiDAR, as well as the quadruped robot perception and motion control platform. In this study, Autoware [17] is used to calibrate the internal parameters of the camera and the external parameters of the camera-LiDAR. It also completes the joint calibration of the depth camera and the LiDAR.

The hardware structure of the quadruped robot perception platform is shown in Figure 2, using a Realsence-D435i depth camera, a Velodyne VLP-16 LiDAR, a Dynamixel MX-28AR digital servo, and an industrial computer Nuvo-7160GC with an NVIDIA GTX 1070Ti. The camera can provide RGB and depth images and is suitable for indoor and outdoor, placed above the LiDAR. The field of view of the depth camera is 86${}^{\circ }$–57${}^{\circ }$. The LiDAR adopted in this study has a 360${}^{\circ }$ horizontal field of view, a 30${}^{\circ }$ vertical field of view and an effective detection distance of 100 m. The digital servo can rotate positive and negative horizontally by 90${}^{\circ }$ to expand the view of the process of leader-following and tracking. Nvidia-GTX1060Ti GPU is used to speed up network model loading and training of detection algorithms based on deep learning. These equipment all have better vibration resistance.

The algorithm structure is shown in Figure 2, which includes a visual leader-following way, a leader-following method based on 3D LiDAR, and a multisensor fusion framework based on an Adaptive Federated Filter. The visual leader-following approach is composed of a person detector (SSD), a person tracker (KCF), and a person reidentification (Re-ID) module. The method based on 3D LiDAR consists of point cloud processing, ground segmentation, and human tracking. The LiDAR algorithm detects the leader through the intensity of the point cloud, so the leader must wear clothes made of reflective material for better detection. The distance between leader and the robot should be kept between 1m and 6m with good tracking performance. The Day/night leader-following method remains effective to detect more distant leader when the distance is within 20 m during day or within 12 m at night.

**Figure 3:**
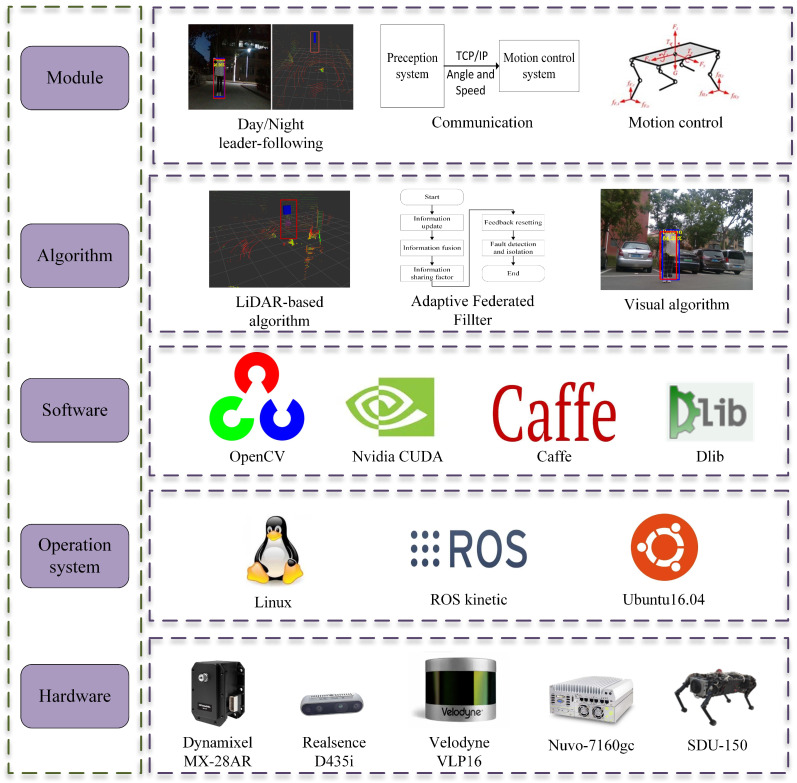
The day/night leader-following system, including application, algorithm, software, operating system, and hardware.

Figure 3 shows the framework of the leader-following system based on multisensor fusion for quadruped robots in day and night. The operating system is Ubuntu 16.04, and the communication among modules is implemented with Robot System Operation (ROS). Additionally, the software used includes OpenCV, CUDA, Caffe, Dlib, Re-ID, etc. These software platforms provide related functions and interfaces to enhance the efficiency of algorithm implementation. The quadruped robot motion control method moves toward the navigator to achieve the task of following the navigator. The leader-following module sends the leader’s angle and distance to the motion control module of the quadruped robots.

### 4.2. Information Sharing Factor Construct

The information sharing factor is important in feeding back the information to the subfilters. Our method makes the information sharing factor change adaptively according to different environments to achieve a better fusion effect.

**Figure 4:**
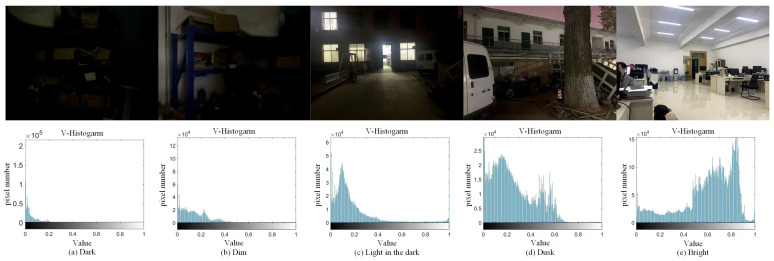
The distribution of value under different lighting conditions, including (**a**) dark, (**b**) dim, (**c**) light in the dark, (**d**) dusk, and (**e**) bright.

This study’s lighting conditions are judged on the basis of the HSV color space. We tested the value distribution under different lighting conditions in Figure 4. The distribution of each pixel value is between 0 and 1, where the median of the value of all pixels can effectively distinguish the lighting condition. As shown in Table 1, we divide the threshold and set the corresponding visual information sharing factor by experiments.

### 4.3. Effectiveness Verification

**Figure 5:**
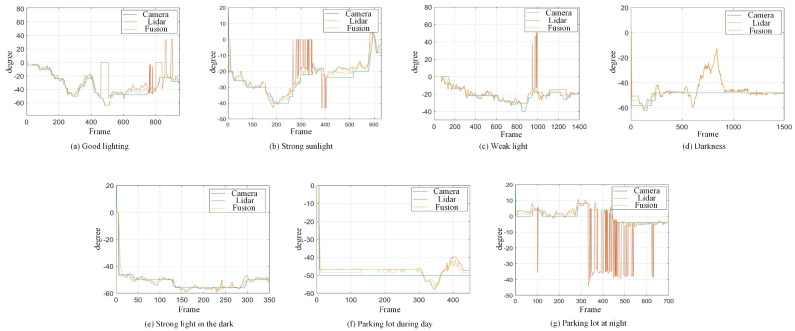
The result of leader-following under different environments, including (**a**) good lighting, (**b**) strong sunlight, (**c**) weak light, (**d**) darkness, (**e**) strong flashlight in the dark, (**f**) parking lot during day, (**g**) parking lot at night.

When the quadruped robot marches, it must face various lighting environments. Therefore, our proposed method’s performance is tested under different lighting conditions. An experiment is conducted on the campus of SDU, where there are many challenges. Table 2 demonstrates the results of our detection algorithm. The first column and the second column in Table 2 show the experimental environment, including good light during the day, strong sunlight at noon, light from streetlights in the evening, total darkness at night, strong flashlight in the dark, parking lot with many license plates made of reflective materials during the day, and a parking lot at night. The third column in Table 2 displays the result of the visual detection algorithm, where the blue detection box is the result of SSD and the red detection box is the result of KCF and Re-ID. The fourth column in Table 2 demonstrates the result of the LiDAR-based detection algorithm, where the blue marker box indicates that the leader is detected. Figure 5 depicts the curves of results of person detection, where the blue curve is the result of visual leader detection, the red curve is the result of LiDAR-based leader detection, and the yellow curve is the result of fusion.

Our method is less disturbed by pedestrians and quadruped robot will continuously following the leader at a speed of 0.5 m/s. When a pedestrian or the leader is detected within 1m of the quadruped robot, the quadruped robot will stop moving forward until the person leaves for safety. Considering the robot’s hardware, if we hope the robot walks 2 μm with a deviation of less than 0.05 μm, the error of leader detection should be less than 3${}^{\circ }$. Therefore, the error in detection within 3${}^{\circ }$ is acceptable.

The experimental result with good lighting is shown in Figure 5a. The leader-following approach proposed achieves a good performance. It is seen that at the frames 450–500, the leader-following method based on LiDAR has a detection error, because the reflective area of cloth decreases when the leader turns. At frame 750, the distance of the leader is so far that the reflection intensity of the point cloud decreases, which leads to the failure of LiDAR detection. At this time, the fusion module detects and handles the errors and obtains a correct person detection result. As mentioned above, the method based on LiDAR will fail when the reflective intensity of clothing on the leader is insufficient. Our proposed approach can detect this failure and handle it. As shown in Figure 5a, the result of the fusion is consistent with the actual position of the leader, and the error with the visual leader-following does not exceed 2${}^{\circ }$. The proposed approach works well.

The experimental result with strong sunlight is shown in Figure 5b. In this experiment, the camera is facing the sun directly. The visual approach is sensitive to bright light, affecting person detection performance. At the frames 0–600, the result of the visual approach is in the shape of a segmented line, which means that visual leader-following lags behind the actual location of the leader. The method based on LiDAR has repeated target loss at frame 300 and multiple detection errors at 400 frames.

Figure 5c is an experimental result with weak sunlight. The experiment is carried out under the condition of only street light illumination at dusk, and the lack of light also affects visual person detection. During the whole process, the result of the visual method also presents a segmented line shape. The visual method temporarily loses the detection target during 400–600 frames. At frames 900 and 1500, the approach based on LiDAR has detection errors. The fault detection system handles the above errors, and the error is less than 3${}^{\circ }$. When the vision sensor faces strong light or weak light, there will be a detection lag and temporary target loss, but multisensor fusion can alleviate this problem. The result of the fusion is consistent with the actual position of the leader in Figure 5b,c, and the value when the single sensor algorithm detects a person correctly, with an error of no more than 2${}^{\circ }$. The proposed approach still works well.

Figure 5d introduces the result of leader-following under darkness. The intelligent quadruped robot must perform tasks at night for practical application, so the ability to stably detect persons in the dark is important. The experiment is conducted indoors at night. It detects the failure of the visual approach and isolates the fault. The result of the fusion is consistent with the actual position of the leader in Figure 5d and fits the result of the LiDAR method. When a single sensor faces a dark environment, detection failure will occur. However, the multisensor fusion and the fault detection based on multisensors information can quickly adapt to the darkness and realize stable leader-following in day and night.

However, the multisensor fusion and the fault detection based on multisensors information can quickly adapt to the darkness and realize stable leader-following in the day and at night. The approach we proposed still works well in the dark. When the quadruped robot walks at night, it may encounter distant car light, which interferes with the person detection, so it is necessary to test our method in the dark with a strong light. Figure 5e is the result of leader-following in the indoor dark with strong light. As shown in Figure 5e, the person moves, the visual detection result remains unchanged, and there is a detection error during frames 0–450. LiDAR is less restricted by light and works well. Our method can use multisensors information to effectively detect visual faults and quickly adapt to darkness with strong light, achieving robust leader-following day and night. The experiment shows that the result of the fusion is within 2${}^{\circ }$ of the actual position of the leader, and our method can stably detect persons in the darkness with a strong light.

Strong reflective materials often interfere with leader-following based on LiDAR, resulting in leader-following errors. Since the license plate is coated with a lot of strong reflective paint, we experimented with reflective interference in a full-car parking lot. Figure 5e,f show the results of leader-following under strong reflection interference during the day and night, respectively. LiDAR is more susceptible to reflective materials such as a license plate in the evening than in the daytime. At 300–500 frames, the method based LiDAR fails, which suffers from repeated target loss and detection errors. The proposed approach detects and fixes the fault so that the error between the result of fusion and that of a working sensor is stable to within 3${}^{\circ }$. Our approach is still effective in the presence of reflective materials.

## 5. Conclusions

In this study, we construct a day/night leader-following method based on an Adaptive Federated Filter algorithm for quadruped robots. This method improves the fault tolerance of leader-following through multisensors fusion and works well in a dark environment without LED light. Specifically, the framework uses a Kalman filter and can adaptively adjust the information sharing factors according to HSV color space, improving stability and robustness under different lighting environments. Additionally, this approach uses multisensors information for fault detection and isolation to solve the problem of various disturbances in complex environments. However, the visual-based approach does not work well under various lighting conditions. The LiDAR-based algorithm will fail when reflective materials are present. We construct an Adaptive Federated Filter algorithm framework to integrate the visual algorithm-based SSD and the LiDAR algorithm-based reflective intensity. The approach can handle the above situations effectively and has strong robustness and fault tolerance. Finally, we achieve reliable and stable leader-following on the quadruped robot SDU-150 in day and night.

Our approach is implemented through decision-level multisensor fusion, which improves the fault tolerance and robustness of the leader-following system but does not improve the accuracy significantly. The next step will combine sensor fusion with deep learning to perform data-level multisensor fusion, which greatly improves the detection accuracy and adapts to the high-precision operating situation.

## Figures and Tables

**Table 1:** Setting of information sharing factor under different lighting.

Median of Value	Lighting Condition	Visual Information Sharing Factor
0–0.2	darkness	0.9
0.2–0.4	weak light	0.7
0.4–1	good light	0.5

**Table 2:** The leader detection result under different conditions.

Light Condition	Experimental Scenes	Visual Detection	LiDAR-Based Detection
Good light	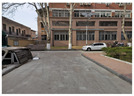	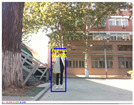	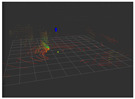
Strong sunlight	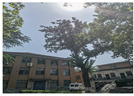	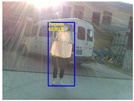	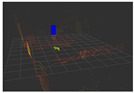
Weak light	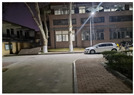	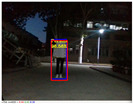	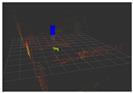
Darkness	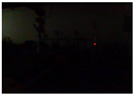	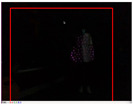	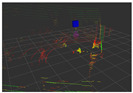
Strong flashlight in the dark	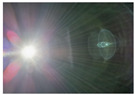	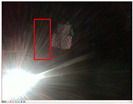	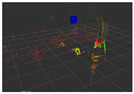
Parking lot during day	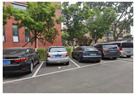	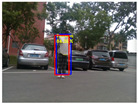	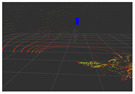
Parking lot at night	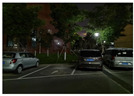	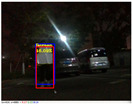	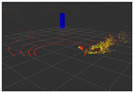

## Data Availability

Not applicable.
